# High-Density μLED-Based Optical Cochlear Implant With Improved Thermomechanical Behavior

**DOI:** 10.3389/fnins.2018.00659

**Published:** 2018-10-01

**Authors:** Eric Klein, Christian Gossler, Oliver Paul, Patrick Ruther

**Affiliations:** ^1^Department of Microsystems Engineering (IMTEK), University of Freiburg, Freiburg, Germany; ^2^BrainLinks-BrainTools, Cluster of Excellence, University of Freiburg, Freiburg, Germany

**Keywords:** cochlear implant, optogenetics, micro light-emitting diode (μLED), thermomechanical behavior, epoxy

## Abstract

This study reports the realization of an optical cochlear implant (oCI) with optimized thermomechanical properties for optogenetic experiments. The oCI probe comprises 144 miniaturized light-emitting diodes (μLEDs) distributed along a bendable, 1.5-cm-long, 350-μm-wide and 26-μm-thick probe shaft, individually controlled via a n × p matrix interconnection. In contrast to our earlier approach based on polyimide (PI) and epoxy resin with different thermal expansion coefficients, the μLEDs and interconnecting wires are now embedded into a triple-layer stack of a single, biocompatible, and highly transparent epoxy material. The new material combination results in a pronounced reduction of thermomechanical bending in comparison with the material pair of the earlier approach. We developed a spin-coating process enabling epoxy resin layers down to 5 μm at thickness variations of less than 7% across the entire carrier wafer. We observed that the cross-linking of epoxy resin layers strongly depends on the spin-coating parameters which were found to be correlated to a potential separation of epoxy resin components of different densities. Furthermore, various metallization layers and corresponding adhesion promoting layers were investigated. We identified the combination of silicon carbide with a titanium-based metallization to provide the highest peeling strength, achieving an adhesion to epoxy improved by a factor of two. In order to obtain a high process yield, we established a stress-free implant release using the electrochemical dissolution of a sacrificial aluminum layer. The direct comparison of oCI probe variants using a single epoxy material and the combination of PI and epoxy resin revealed that the epoxy-resin-only probe shows minimal thermomechanical probe bending with a negligible hysteresis. The thermal probe characterization demonstrated that the temperature increase is limited to 1 K at μLED DC currents of up to 10 mA depending on the stimulation duration and the medium surrounding the probe. The optical output power and peak wavelengths of the new oCI variant were extracted to be 0.82 mW and 462 nm when operating the μLEDs at 10 mA, 10 kHz, and a duty cycle of 10%. The optical power corresponds to a radiant emittance of 407 mW/mm^2^, sufficient for optogenetic experiments using channelrhodopsin-2.

## Introduction

With more than 400,000 implanted systems, cochlear implants (CIs) currently represent the most successful neuroprosthetic device (Zeng and Canlon, 2015). In general, CIs comprise up to 22 electrodes arranged along a slender implantable probe with a typical length of up to 30 mm and diameters between 0.25 (distal end) and 0.6 mm (apical position). They are implanted into the scala tympani of the cochlea through the round window and are used to electrically stimulate the spiral ganglion neurons (SGNs) to partially restore hearing. Most CIs are implanted in very young children starting at the age of 6 months as they learn much faster to handle the electrical signals produced by a CI, which improves the early development of their hearing and speaking capabilities (Conner et al., 2006). Nonetheless, older children and adults, suffering from a sudden hearing loss, receive CIs as well. However, despite hearing restoration, both groups of patients encounter the main drawback of classical CIs, which is the limited frequency resolution as a consequence of the wide current spreading in the scala tympani (Kral et al., 1998; Wheeler et al., 2007; Zeng et al., 2008). This restriction in terms of frequency resolution is taken into account by the stimulation algorithms of modern CIs, offering patients a reasonable comprehension of speech (Zeng et al., 2008). Although improvements have been achieved with advanced stimulation techniques (Zhu et al., 2012), even children with early implantations and therefore a good result of hearing restoration are rarely able to conduct conversations in noisy environments or to appreciate music.

A promising approach to overcome the limitations of classical CIs is offered by optogenetics (Jeschke and Moser, 2015). Optogenetics enables the direct interaction with neurons using light-sensitive opsins integrated into the neuronal cell membranes (Deisseroth et al., 2006; Yizhar et al., 2011). The most widely used opsin is channelrhodopsin-2 (ChR2) with a peak sensitivity at 470 nm (Pashaie et al., 2014) matching the emission spectra of highly efficient gallium nitride (GaN) light-emitting diodes (LED). CIs designed for optical stimulation have been proposed to integrate linear arrays of micro LEDs (μLEDs) on a slender, bendable probe allowing the direct optical stimulation of the SGNs (Goßler et al., 2014). This approach of a so-called optical CI (oCI) promises neuronal stimulation with improved spatial resolution, thereby improving the frequency decoding by a factor of 10 depending on the number of applicable μLEDs and on the cellular selectivity.

Several approaches for a controlled delivery of light into neuronal tissue have been previously described. Often, the technical interfaces have taken advantage of optical glass fibers (Aravanis et al., 2007; Zhang et al., 2007; Pisanello et al., 2017) guiding the light from an external light source, e.g., a solid state laser or a high-power LED, to the area of interest. This approach has the advantage that light with a broad range of wavelengths matching the sensitivity spectrum of various opsins can be delivered. However, there are several reasons why optical fibers are far from optimal as an oCI: a first reason is the need for an external light source; secondly, the mechanical stiffness of the optical glass fibers is hardly compatible with the bending radii of the cochlea; thirdly, even if the bending problem was solved, the internal reflection needed for light guiding is strongly reduced in bent fibers. The direct integration of laser diode (LD) chips with light guiding structures, as described in Park et al. (2011), Kampasi et al. (2016), and Schwaerzle et al. (2017), overcomes at least the size constraints of an external light source. However, it imposes restrictions in terms of applicable wavelengths due to the limited commercial availability of compact, unpackaged LD chips.

A more promising approach is based on the integration of LEDs either as bare LED chips (Kwon et al., 2013a; Ayub et al., 2017; Ji et al., 2017) or as thin-film μLEDs (Goßler et al., 2014; Hahn et al., 2014; Wu et al., 2015; Ayub et al., 2016; Klein et al., 2016; Scharf et al., 2016) on the implantable probe. In the case of bare LED chips, the probe design needs to fit to the size of available chips with typical lateral dimensions of 220 × 270 μm^2^ and a thickness of 50 μm or more (Schwaerzle et al., 2016). The integration of these LED chips has been achieved using wire bonding and adhesive fixation (Ji et al., 2017), which has been accompanied by a pronounced size increase due to the wire bonds. Alternatively, flip-chip bonding (Ayub et al., 2016; Schwaerzle et al., 2016) on either flexible or stiff substrates has enabled smaller system dimensions limited only by the size of the LED chips. As an example, Schwaerzle et al. (2016) presented an oCI based on 10 individually controllable LED chips on a custom-made polyimide (PI) carrier substrate; with this system, the optical evocation of auditory brainstem responses (ABRs) has been demonstrated. Nevertheless, the size of commercially available LED chips has remained an obstacle to higher lateral resolutions in LED arrangement and a reduction of the implant bending radii required for reaching deeper positions in the cochlea (Jeschke and Moser, 2015).

The above limitations can be circumvented by the approach of Goßler et al. (2014) integrating linear μLED arrays on flexible PI substrates using a wafer-level laser lift-off (LLO) transfer of the μLEDs. As a result, the oCI width was successfully reduced to 380 μm and 15 μLEDs with a size of 150 × 150 μm^2^ were integrated on the probe. The μLEDs were controlled in groups of five via three individually addressable electrical channels. The wafer-level transfer and assembly process enable hundreds of these μLEDs of minimal lateral dimensions to be handled in parallel, which could not realistically be achieved by flip-chip bonding. The fabrication process of this oCI variant relies on different polymers, i.e., PI and epoxy resin, serving as the substrate and for encapsulation, respectively. The mismatch in the coefficients of thermal expansion (CTEs) of the two polymers caused a pronounced mechanical bending of the oCI upon temperature changes during probe processing and encapsulation or simply by activating the μLEDs. This bending made implantation into the windings of the cochlea difficult because the implant was curled in the wrong direction. Finally, the mismatch in the CTEs leads to mechanical stress within the implant itself which potentially causes delamination between the polymer layers and thus the failure of the oCI.

The study presented here addresses in particular the thermomechanical behavior of the oCI by replacing its PI substrate by an epoxy layer. This is the same material that is already used as an underfill during the LLO process and for the encapsulation of the top n-contact metallization. In comparison to the state of the art, the study demonstrates a pronounced increase in the number of integrated μLEDs while reducing the overall probe width. The thermomechanical behavior of the new oCI variant is successfully improved over that of the hybrid PI/epoxy probe. Aside from the optical probe characterization, i.e., μLED center wavelength and radiant flux, the study further evaluates the temperature increase on the probe surface due to μLED operation which needs to be limited to 1 K for a safe *in vivo* probe application.

## Materials and Methods

### Design of the oCI

**FIGURE 1:**
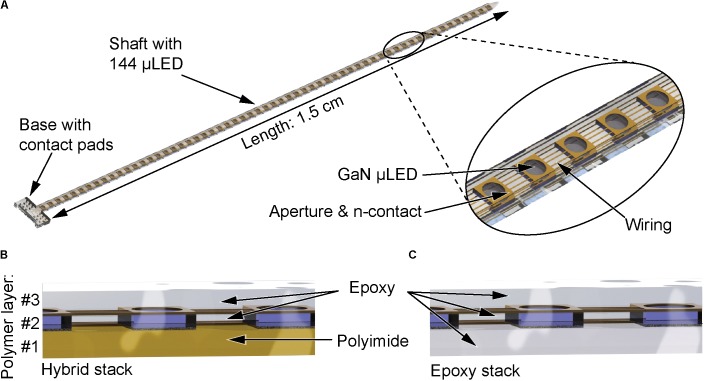
**(A)** Schematic of the oCI probe comprising a probe base carrying contact pads and a 1.5-cm-long shaft comprising 144 μLEDs. The μLEDs are contacted by square metal pads serving as n-contacts, with the emitting areas confined by apertures with a diameter of 50 μm. Cross-section **(B)** shows the state-of-the-art hybrid polymer stack with a combination of PI and epoxy resin layers. Cross-section **(C)** shows the novel polymer stack making exclusive use of epoxy resin layers.

The oCI developed in this study is schematically shown in **Figure 1A**. It comprises 144 individually addressable μLEDs controlled via 12 n-contact and 12 p-contact pads using a 12×12 matrix interconnection scheme. The contact pads are located on the oCI base with in-plane dimensions of 1500 × 680 μm^2^. The top n-metallization of the μLED comprises a circular aperture with a diameter of 50 μm confining the light emission from the μLED. The μLEDs are distributed along the 1.5-cm-long, 350-μm-wide, and 26-μm-thick flexible oCI shaft at a pitch of 100 μm. The oCI metallization interfacing the n- and p-doped GaN of the μLEDs is embedded in a three-layer polymer stack whose individual components each serve a specific task. The bottom layer (layer #1) represents the substrate comprising the p-contact metallization onto which the μLEDs are transferred using the LLO process. The second polymer layer (layer #2) plays the role of an underfill during the LLO process and electrically insulates the n-contact and p-contact metallizations from each other. Finally, the third polymer (layer #3) serves as the passivation layer of the n-contact metallization. Obviously, these layers need to offer process compatibility and biocompatibility as well as a high optical transparency at the peak emission wavelength of 462 nm of the applied GaN μLEDs. Two oCI variants, i.e., the hybrid oCI, as introduced by Goßler et al. (2014), and the epoxy-resin-only variant developed here, are analyzed in this study. In the case of the hybrid oCI, a stack of one PI and two epoxy resin thin films is used for layers #1 to #3, as illustrated with the cross-section in **Figure 1B**. More specifically, these are the PI U-Varnish-S (UBE Industries Ltd., United States) and the epoxy resin E301 (Epoxy-Technologies, United States). In contrast, the novel epoxy-resin-only oCI comprises three layers of E301 (**Figure 1C**). As the CTEs of the used PI and epoxy resin are mismatched by a factor of 13 (see **Table 1**; Epoxy Technology; UBE Industries Ltd., United States^1^), a strong residual bending of the oCI probes has resulted in the hybrid probe. This was observed following oCI processing and thermal cycling, i.e., by operating the μLEDs and locally heating the oCI. By replacing the PI substrate, the mechanical structure of the oCI is expected to benefit of an improved thermomechanical homogeneity minimizing the residual probe bending.

**Table 1:** Physical properties of the polymers polyimide U-Varnish-S and epoxy resin E301 used to realize oCI probes.

Material	Transmission @ 470 nm	Curing temperature	Bio-compatibility	Peel-off from SiO_2_	Solvent	CTE α_th_ (10^-6^ K^-1^)
U-Varnish-S (UBE Industries Ltd., United States)	25–35%	450°C	Not specified	Possible	NMP	3
E301 (Epoxy Technology)	>99%	80°C	Yes	Not possible	None	38


### Materials

The oCI combines components realized using the materials listed in **Table 2** indicating the respective material functionalities. As described in further detail in the section “Wafer Bonding Process”, μLEDs are transferred from a sapphire fabrication wafer to a polymer substrate comprising the p-contact metallization using epoxy resin as an underfill. It serves the purpose of mechanically stabilizing the μLEDs during the LLO process. The n-contact metallization, which is processed on the underfill after LLO, is subsequently passivated by another epoxy resin layer.

**Table 2:** Materials applied during oCI fabrication.

Material	Function	Thickness	Deposition
Silicon (Si)	Carrier substrate initially used for the polymer substrates	525 μm	–
Sapphire	Carrier substrate of μLEDs and polymer substrate	626 μm	–
Epoxy resin E301 (E301)	oCI substrate Underfill Passivation	10 μm 6 μm 10 μm	Spin-coating Dip-coating Spin-coating
Polyimide (PI)	oCI substrate	5 μm	Spin-coating
Gallium nitride (GaN)	μLED	6 μm	Epitaxial growth
Silicon oxide (SiO*_x_*)	To facilitate PI release	200 nm	PECVD
Silicon nitride (Si*_x_*N*_y_*)	Passivation of GaN mesas and protective film for underfill		PECVD
Aluminum (Al)	Sacrificial layer	1 μm	Sputter deposition
Gold (Au)	p-contact Metal tracks	5 nm 400 nm	Evaporation Sputter deposition
Indium (In)	Bonding metal	4 μm	Evaporation
Tungsten-titanium (WTi)	Diffusion barrier Contact layer during Al dissolution	200 nm 200 nm	Sputter deposition
Platinum (Pt)	Diffusion barrier Adhesion promoter	50 nm 40 nm	Sputter deposition
Silver (Ag)	Reflective p-contact	100 nm	Evaporation
Nickel (Ni)	Formation and adhesion of p-contact	5 nm	Evaporation
Titanium (Ti)	Adhesion promoter Diffusion barrier	40 nm	Sputter deposition


The basic process compatibility of the oCI materials has already been demonstrated in previous work: (i) μLEDs have been realized on sapphire wafers (Goßler et al., 2014; Ayub et al., 2016; Klein et al., 2016), (ii) indium (In) has been deposited as a bonding material on μLED mesas (Klein et al., 2016), and (iii) the μLEDs have been transferred wafer-wise onto polymer (Goßler et al., 2014) and silicon (Si) (Ayub et al., 2016) substrates using LLO and epoxy resin underfill. In order to improve the thermomechanical behavior of the oCI probes, the PI substrate comprising the p-contact metallization originally processed on Si carrier wafers had to be replaced by an epoxy resin layer. For this purpose, some fabrication steps needed to be developed. They targeted the spin-coating of the epoxy resin serving as polymer layer #1 and its release from a carrier substrate. In contrast to PI, which is easily peeled off a silicon oxide (SiO_2_) naturally grown on silicon (Si) carrier wafers (Goßler et al., 2014), sapphire carrier substrates will be introduced in the newly established process. The main reason behind this additional change in oCI processing is the residual mechanical stress which strongly affects the wafer-level μLED bonding. This is in particular the case when μLEDs realized on sapphire are transferred onto Si substrates. Again, the mechanical stress is caused by differences in the CTEs, in this case those of sapphire and Si, as discussed in detail in Klein et al. (2016).

These new material modifications required the following process developments and optimizations:

• spin-coating of a solvent-free epoxy resin film to achieve thin polymer layers with homogeneous thicknesses down to 5 μm enabling a complete layer curing;
• layer adhesion between the epoxy resin layer and the gold-based oCI p-contact metallization;
• probe release from the carrier substrate exerting minimal mechanical stress on the oCI probes.

Material properties of layers #2 and #3 are subject to some requirements in view of oCI processing and probe functionality. These are

• that the epoxy resin of polymer layer #2, i.e., of the underfill, be solvent-free and cure at temperatures below 170°C with minimal material shrinkage. The first request is due to the fact that the material needs to be cured in the narrow gap between the sapphire-carrying μLED wafer and the carrier wafer of the polymer substrate, where the exchange of solvents by diffusion is limited; the requirement about curing temperature and shrinkage aims at ensuring the integrity of the gold (Au)–In-based bond between the μLED and oCI p-contact metallization;
• that the polymer layer #3, i.e., the probe passivation, be highly transparent at the μLED wavelength around 460 nm and fulfill the same restrictions regarding thermal budget.

Upon considering these requirements, we chose the epoxy resin E301 as the material of choice since it is optically transparent and cures below 170°C, the critical temperature for the μLED bond. The used polymers in the oCI and their process behaviors are summarized in **Table 1**.

### oCI Fabrication

**FIGURE 2:**
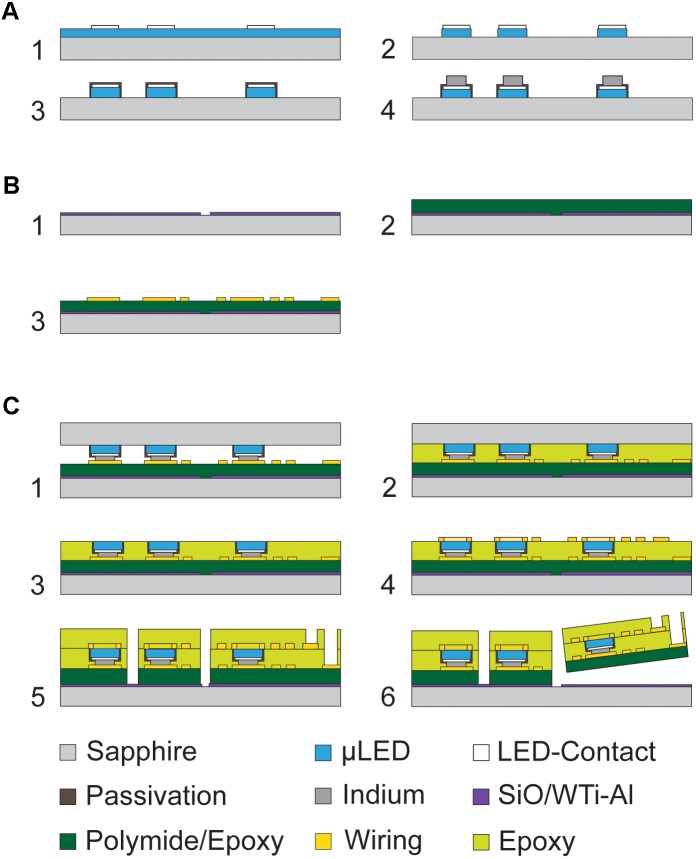
**(A)** Main steps of the wafer-level μLED fabrication process including the deposition of the μLED metallization, etching of GaN, deposition of Si*_x_*N*_y_* passivation and contact metallization; **(B)** main steps of the carrier wafer fabrication; **(C)** main steps of the oCI process based on the wafer-level transfer of μLEDs to the polymer substrate supported by a carrier wafer.

A schematic of the oCI manufacturing process is shown in **Figure 2**. This wafer-level process can be split into the fabrication sequences of (i) the μLEDs on a sapphire wafer (**Figure 2A**), (ii) the polymer substrate with p-contact metallization (**Figure 2B**), and (iii) the μLED transfer and bonding with subsequent n-contact metallization and passivation (**Figure 2C**). The μLED process applies epitaxial growth of GaN by metalorganic vapor phase epitaxy and needs to be performed on a sapphire substrate at temperatures above 1000°C to achieve high LED efficiencies (Nakamura, 1991). Although the polymer substrate is processed on a carrier wafer using moderate temperatures, i.e., max. 450°C, sapphire is used as well in order to minimize the thermomechanical stress during the wafer-level bonding that had affected the structures in the case of Si substrate wafers (Klein et al., 2016).

#### GaN-Based μLED Process

The μLED process applies commercial GaN-on-sapphire wafers. It starts with cleaning the GaN surface by dipping the wafers for 5 min into 5 vol.% hydrochloric acid (HCl) followed by the first photolithography step using an image reversal photoresist (AZ5214E, Microchemicals, Ulm, Germany). Then 5 nm of nickel (Ni) and 5 nm of Au are deposited by evaporation in order to create the first layer of the p-contact metallization of the p-doped GaN. Following resist lift-off, the p-contacts are annealed in oxygen atmosphere at 550°C to lower the contact resistance; at this step, a Ni oxide forms at the surface. The Ni oxide is subsequently removed by oxalic acid to achieve an interface with low contact resistance (Mengzhe et al., 2009) and high optical transmittance through this first layer of the p-contact. Using the same photolithography mask and AZ5214E photoresist, 100 nm of silver (Ag) is evaporated onto the preprocessed p-contact to form the reflective part of the p-contact. A second annealing step in nitrogen (N_2_) atmosphere performed at 400°C forms the final contact with a reflectivity of 81% at 470 nm and a contact resistance of 2.9 × 10^-4^ Ωcm^2^.

Next, a diffusion barrier is deposited onto the Ag-based p-contact metallization. It serves as a protection of the p-contact and underlying GaN against In diffusion. The In is later used as a bond metal. The diffusion barrier comprises 200 nm of tungsten titanium (WTi; composition of 90% W and 10% Ti) and 50 nm of platinum (Pt) as an adhesion promoter for the In bond metal. The p-contact and the diffusion barrier are schematically shown in **Figure 2A1**.

A photoresist masking layer (AZ9260, Microchemicals, Ulm, Germany) with a thickness of 15 μm is used to pattern the GaN layer in a chlorine-based plasma etch process and to form the μLEDs structures. The etch process is performed in an inductively coupled plasma etcher at a reduced power of 170 W, resulting in an etch rate of 660 nm/min. This moderate etch rate avoids high temperatures and maintains the photoresist integrity. Following the resist strip, 6-μm-high GaN mesas structures with p-contacts are obtained, as schematically shown in **Figure 2A2**.

The GaN μLED mesas are then passivated with a layer of silicon nitride (Si*_x_*N*_y_*, 1 μm) applied by plasma-enhanced chemical vapor deposition (PECVD). This layer serves as a protective film of the μLED sidewalls against short circuits. The Si*_x_*N*_y_* on the sapphire surface between the μLED mesas is structured using a 10-μm-thick AZ9260 photoresist mask and reactive ion etching (RIE). As a result, only the μLED mesas and their respective metallization are covered with Si*_x_*N*_y_* (**Figure 2A3**). The exposed sapphire substrate between the GaN mesas and the μLED structures is next covered by an additional 50-nm-thin PECVD Si*_x_*N*_y_* layer. It is later needed as a protective film against the polymer underfill used during the LLO process.

In order to prepare the μLED wafer for bonding onto the polymer substrate on its carrier wafer, a 4-μm-thick In layer is deposited on the 6-μm-high μLED mesas. The In patterns are defined using a bi-layer lift-off process applying a 12-μm-high layer of LOR 30B photoresist (MicroChem, MA, United States) and a 7-μm-thick AZ4533 photoresist (Microchemicals, Ulm, Germany). With this protection in place, the Si*_x_*N*_y_* passivation on the μLED p-contact and diffusion barrier is then opened using RIE. Subsequently, the In bond layer is evaporated, as described in Klein et al. (2016), and patterned by lifting off the bi-layer photoresist stack to finalize the μLED process (**Figure 2A4**).

#### Polymer Substrate Process

The process sequence to realize the polymer substrates of the oCIs is illustrated in **Figure 2B**. In contrast to the state-of-the-art hybrid oCIs (Goßler et al., 2014), in this study the process is performed on sapphire wafers to avoid thermal stress during the In-based wafer bonding, which had hampered earlier systems (Klein et al., 2016). In order to compare both oCI variants, i.e., the hybrid and epoxy-resin-only structures, we realized two polymer substrate variants, as described in detail in the following sections.

##### PI-based oCI probes

In the case of PI, a 200-nm-thin PECVD SiO*_x_* layer is deposited on the sapphire carrier wafer in order to enable the final probe peel-off. This oxide layer is structured using a photoresist layer (AZ1518, Microchemicals, Ulm, Germany) and RIE such that SiO*_x_* remains only in those wafer areas where the implants are realized (**Figure 2B1**). In the other areas the sapphire surface therefore remains exposed. This ensures that the PI film optimally adheres directly to the carrier wafer minimizing the effect of a potential PI delamination on the implants, thus increasing the process control and fabrication yield.

Following the carrier wafer preparation, PI is spin-coated to a thickness of 5 μm and then cured in N_2_ atmosphere at a peak temperature of 450°C (**Figure 2B2**). This is followed by the deposition of the p-side metallization of the μLEDs using a bi-layer lift-off process applying a 1-μm-thin lift-off resist (LOR10B, MicroChem, MA, United States) in combination with a 3-μm-thick positive photoresist (AZ1518). Prior to the metal deposition, the PI surface is cleaned and activated in argon (Ar) plasma followed by the sputter-deposition of 40 nm of Pt, 400 nm of Au, and 40 nm of Ti without breaking the vacuum. The Pt film serves as the adhesion promoter to PI while Au is the highly conductive part of the metal stack. In the subsequent wafer-level bonding process, the top Ti film prohibits the diffusion of In along the metal tracks. The lift-off is performed using a lift-off tool applying dimethyl sulfoxide with high-pressure to the wafer surface. Subsequently, the wafer is rinsed to ensure a clean substrate surface. In the final step of the polymer substrate fabrication, the Ti layer is removed on the contact pads in order to enable the wafer-level bonding by In–Au reflow. This Ti etch step is performed with a 4-μm-thick photoresist (AZ4533) using hydrofluoric acid (HF, 1%) for 15 s to expose the Au underneath (**Figure 2B3**).

##### Epoxy-resin-only oCI probes

In the case of the epoxy-resin-based oCI substrates, the carrier wafer has to be equipped with a sacrificial layer as epoxy resin can neither be peeled from the sapphire substrate nor from the SiO*_x_* layer used in the case of the PI substrates. We addressed this challenge by applying a sacrificial aluminum (Al) layer which is later dissolved by anodic metal dissolution (Metz et al., 2005) for oCI probe release. This Al film is deposited on an additional, conductive WTi layer which remains unaffected by the sacrificial Al removal. This WTi layer is necessary to guarantee electrical contact throughout the entire release and thus ensures the complete Al removal. We applied a 200-nm-thin WTi film and a 1-μm-thick Al layer which are sputter-deposited and evaporated, respectively. This layer stack is then structured into areas of 1 × 2 cm^2^ enabling the local lift-off of 12 oCIs per area. The masking is done using the AZ4533 photoresist followed by selective etching of Al and WTi at 50°C using a commercial Al-etchant [mixture of phosphoric, nitric, and acetic acids diluted in water at a ratio of 12:3:1:3 (Westberg et al., 1996)] and hydrogen peroxide (H_2_O_2_, 20 wt.%), respectively.

*Spin-coating* – Following the carrier wafer preparation, the epoxy resin E301 needs to be deposited with homogenous layer thickness. Similar to PI, spin-coating was chosen as the deposition method. As the E301 resin consists of two components that have to be mixed prior to layer deposition and as it does not contain any solvent, spin-coating this material presents several challenges compared to others such as photoresist or the solvent-based epoxy resin SU-8.

First, the E301 layer does not dry during spin-coating like solvent-based films. Furthermore, it has a low interfacial energy with sapphire, which leads to the formation of thicker islands rather than a homogeneous layer. Island formation most often starts at the edges and the middle of the carrier wafer. In order to suppress this effect, the adhesion promoter Protek B3 Prime (Brewer Science, Rolla, MO, United States) is applied; it basically provides a surface silanization. We use a dehydration bake (205°C, 5 min) and a primer baking step (205°C, 1 min) between which the adhesion promoter is spin-coated. Spin-coating E301 on a primed wafer shows a much improved wetting as a consequence of which the E301 no longer collapses into islands. Nevertheless, the material still starts creeping toward the wafer center within a minute after spin-coating, hence reducing the wafer area covered by E301. In order to prevent this undesired effect, the E301-coated wafer needs to be transferred to a hotplate within seconds to initiate the curing procedure in particular at the interface between primer and epoxy resin. We chose a hot plate temperature of 75°C, which is below the evaporation temperature of both E301 components. The curing temperature was kept constant for 3 h; it was then ramped up to 120°C within 10 min; the wafer is kept at that temperature for additional 12 h.

During the process development, we evaluated the effect of the spinning speed (between 1,000 and 7,000 rpm in steps of 1,000 rpm) and spinning duration (between 2 and 30 s) on layer thickness, thickness homogeneity, and material cross-linking.

*Metallization* – In addition to the spin-coating parameters, the adhesion of the subsequently deposited layers needs to be evaluated as well. In the case of the epoxy-resin-based oCI probes, only the adhesion of the p-contact metallization has to be analyzed. This is because the E301 underfill is expected to adhere inseparable to an already cured E301 layer. In this study we investigated layer stacks of chromium Cr/Au/Ti, Pt/Au/Ti, and Ti/Au/Ti where Au (400 nm) serves as the conductive part of the wiring, and the upper Ti layer (40 nm) acts as a diffusion barrier during the subsequent μLED transfer process. For the first metal layer, we tested Pt, Ti, and Cr. These are commonly used to improve the layer adhesion (Vancea et al., 1989; Ordonez et al., 2012a). The metal stacks are sputter-deposited and patterned by the lift-off technique.

As adhesion promoters we tested (i) surface silanization by spin-coating and from the vapor phase at 120°C, (ii) a 20-nm-thin silicon carbide (SiC) film deposited using PECVD at 100°C, and (iii) the chemical treatment of the epoxide functional groups by dipping the epoxy resin layers into 37 wt.% HCl intended to split the epoxy rings into pairs of hydroxyl groups.

#### Wafer Bonding Process

The third sequence of the oCI fabrication process transfers the μLEDs from the sapphire wafer to the polymer substrate supported by its carrier wafer. The respective wafer bond applies an In–Au interdiffusion bonding process (Goßler et al., 2014). For wafer bonding, the μLED wafer is aligned upside down with respect to the Au pads on the polymer substrate using the mask aligner MA/BA6 (Karl Süss, Garching, Germany). The dual wafer sandwich is then transferred to the wafer bonder SB6 (Karl Süss, Garching, Germany) where a pressure of 80 kPa is applied at 140°C for 30 min under vacuum (Klein et al., 2016) [see cross-section in **Figure 2C1**].

The gap between both sapphire wafers mainly resulting from GaN mesas and the In bond metal is filled with an epoxy resin to mechanically stabilize the μLEDs during the subsequent LLO process. The polymeric underfill is performed by exposing the wafer sandwich under vacuum to liquid epoxy resin. Once the wafer has been immersed into the epoxy, a pressure of 1 bar is applied using N_2_ atmosphere. The combination of an initial vacuum followed by applying a pressure facilitates the capillary gap filling **(Figure 2C2)**. In general, only a small void remains at the wafer center. It is usually smaller than 5% of the total wafer area. The epoxy resin is cured at room temperature (RT) for 12 h followed by a temperature ramp to 120°C at which the wafer remains for 12 h. This procedure ensures that none of the epoxy resin components evaporates prior to the completion of their cross-linking.

#### Laser Lift-Off

Next, the μLED sapphire wafer is released in a LLO process using a 248 nm excimer laser (3D-Micromac, Germany, Chemnitz) (Goßler et al., 2014). During the LLO process, the individual μLEDs are delaminated first using a laser fluence of 800 mJ/cm^2^ followed by the delamination of the underfill at 500 mJ/cm^2^. This sequence prevents the fracture of the μLEDs by the compression waves caused by the decomposition of GaN into Ga and N_2_ under the laser light. After the laser has been processed the entire wafer, the sapphire wafer can simply be lifted off using tweezers **(Figure 2C3)**. In preparation of the deposition of the n-side metallization, residual Ga on the μLED surface is removed using a 5-min-long exposure to ammonia (NH_3_, 5 vol.%).

#### Probe Metallization, Patterning, and Release

Similar to the p-side wiring, we apply an Au-based metallization stack, where Ti is applied as the first metal, as it provides a better adhesion to the epoxy underfill. The metalized wafer with μLEDs connected on both sides is shown in **Figure 2C4**. The last layer of epoxy resin is then again applied by spin-coating. The rough surface of the second epoxy layer resulting from the LLO is favorable enough for layer adhesion so that no adhesion promoter is needed. The epoxy resin is cured for 3 h at 75°C followed by 12 h at 120°C. Subsequently, a 30-μm-thick photoresist etch mask (AZ9260) is applied to open the contact pads and to trench the polymer stack by RIE down to the substrate for probe separation **(Figure 2C5)**. The process is similar to that described in Goßler et al. (2014) and Ayub et al. (2016).

In the case where PI is used as the polymer substrate, the oCI probes can be simply peeled off the carrier wafer one by one using tweezers. In the epoxy substrate case, the sacrificial Al layer is removed using anodic metal dissolution, thereby releasing the oCIs (Westberg et al., 1996). To connect the Al layer a spring-loaded needle connects the wafer surface in dedicated etch openings (300×300 μm^2^) between the oCIs. The Al layer is dissolved by immersing the entire wafer into 0.1 M saline solution and applying a voltage of 1.6 V with respect to the solution. The counter electrode is made of a ceramic substrate coated with Pt. The etch openings in the polymer layer provide electrical contact to a field of 12 oCIs which are released from the carrier wafer and are ready to be picked up from the solution using tweezers. In general, the sacrificial layer approach benefits the oCI samples by a pronounced reduction in mechanical stress during the release.

### Process Evaluation and oCI Probe Characterization

#### Characterization of the Spin-Coated Epoxy Resin Layers

The spin-coated epoxy resin thickness was measured using the profilometer P11 (Tencor, Milpitas, CA, United States) as a function of spinning parameters at nine positions across the 4-inch wafers by cutting out squares of the epoxy film with a size of ca. 2 × 2 mm^2^. Aside from the complete, homogenous coverage of the carrier wafer, a complete cross-linking of the spin-coated epoxy resin is crucial. Depending on the spin-coating parameters, we observed that some E301 layers remained sticky on the surface, indicating an incomplete cross-linking of the material. Due to the used deposition process, differential scanning calorimetry, which is widely applied to analyze the cross-linking of epoxide materials (Montserrat et al., 2003) could not be used here. The main reason is that 5 mg of material would be required for such an analysis while spin-coating covers the 4-inch wafers with just 10 μg of epoxy resin. Instead, the etch rate of differently spin-coated E301 layers processed in an oxygen plasma was used as a measure of the level of cross-linking. In order to extract the epoxy resin etch rates and process uniformity across the test wafers, we measured the layer thicknesses *h*_ini_ and *h*_etch_ before and after plasma etching at nine positions, as described above. The etch rates are compared to the etch rate of bulk E301 samples cured under standard conditions to achieve more than 99% cross-linking (Epoxy Technology). All test samples including the bulk sample have been processed on 4-inch wafers representing an etch load above 99% which allows to eliminate the influence of any masking layer. During the etch process the wafers are actively cooled while the wafer temperature is monitored in order to exclude the influence of an increased temperature on the etch rate. It was found that the etch rate decreases with increased level of cross-linking.

#### Metal Layer Adhesion

**FIGURE 3:**
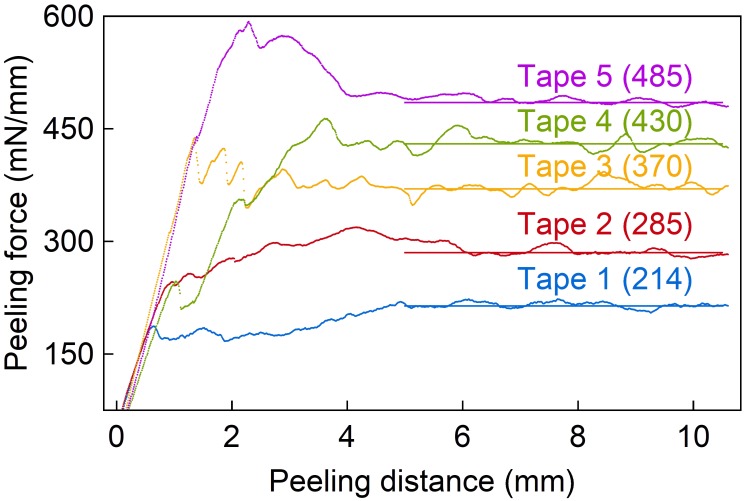
Peeling force normalized to the tape width vs. peeling distance providing a measure of the adhesion strength of the applied adhesive tapes used in the chessboard test. Horizontal lines indicate the values used for the adhesion testing.

The metal layer adhesion to E301 was tested for several adhesion promoters using an extended peel-off test. The adhesive tapes used here have the same carrier material but different adhesion properties. These are first characterized on a sputtered Ti layer representing the uppermost surface of the metal stack to be analyzed (section “Epoxy-resin-only oCI probes”). The adhesive strength between different tapes and the Ti is shown in **Figure 3** with the peeling force per tape width as a function of peeling distance. These measurements indicate a difference in the peeling force by a factor of 2.2 between the weakest and strongest tape.

**FIGURE 4:**
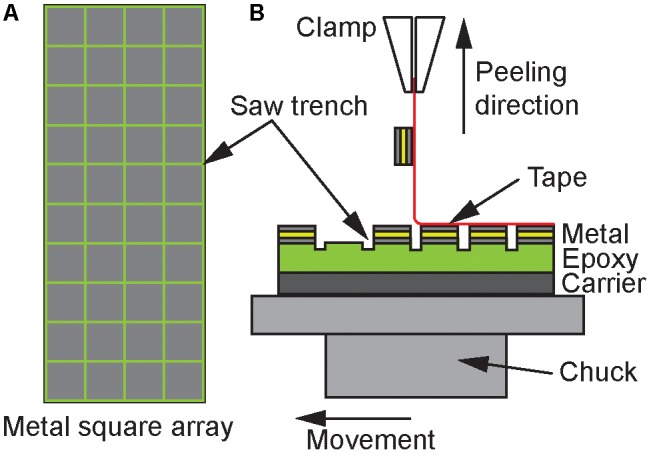
**(A)** Peel-off probe consisting of 2 × 2 mm^2^ squares arranged in a 4 × 10 array. **(B)** Peeling test with different tapes, peeled perpendicular to the probe surface.

The metal layer stack deposited onto the epoxy resin E301 is subdivided into a 4 ×10 array with squares of 2×2 mm^2^ separated by 200-μm-wide trenches realized using a wafer saw that partially cuts into the underling E301 layer. The peeling tests under 90°, as illustrated in **Figure 4**, use different tapes with a width of 12 mm slightly overlapping the array to eliminate edge effects. The adhesive strength of the metal layer stacks is evaluated using the number of metal squares remaining on the wafer after peel-testing with the adhesive tapes of different adhesive strengths.

#### Thermomechanical Behavior

To investigate the thermomechanical behavior of the hybrid and epoxy-resin-only oCI samples, both types of implants have been exposed to a heat ramp from RT to 100°C and subsequently cooling down to RT. For this purpose, the oCI probes are placed in a glass container to minimize any perturbation from air convection and to enable visual observation using a microscope. The glass container is heated using a hot plate while the temperature is monitored by a PT100 temperature sensor. The temperature was raised and reduced in steps of 5 K and kept constant for 2 min to stabilize the temperature of the oCI.

#### Thermal Characterization

The temperature increase on the probe surface was monitored using an infrared camera (PI 450, Optris GmbH, Berlin, Deutschland) while one μLED was operated with DC of up to 10 mA. The probe under test was either floating in air, or immersed into agarose gel (0.5 wt.%, Agar-Agar, Roth, Karlsruhe, Germany) or water both kept at a temperature of 37°C mimicking the body temperature. In the case of agarose gel and water, the probe surface to be measured with the IR camera was covered by 5 μm of the immersion material adjusted using a precise translational stage. The opposite side of the probe was facing the bulk of the immersion material with a thickness of at least 10 mm.

#### Optical Characterization of oCI Probes

The oCI probes are optically characterized using an integration sphere (ISP-50-I-USB, Ocean Optics, Ostfildern, Germany) (Schwaerzle et al., 2017). The probes mounted on a PCB and wire bonded to it are operated with a drive current between 0 and 10 mA while the radiant flux and the spectrum of emitted wavelengths are measured.

## Results and Discussion

### Process Validation

#### Spin-Coating

Spin-coating test series at various spin speeds and spin durations revealed a maximum thickness variation of the E301 epoxy resin of 7%, measured between the wafer center and a radius of 45 mm. In contrast to solvent-based polymer solutions such as PI, this high thickness homogeneity is independent of the spin-coating parameters. It is caused by the fact that E301 is solvent-free and levels itself during the curing procedure.

**FIGURE 5:**
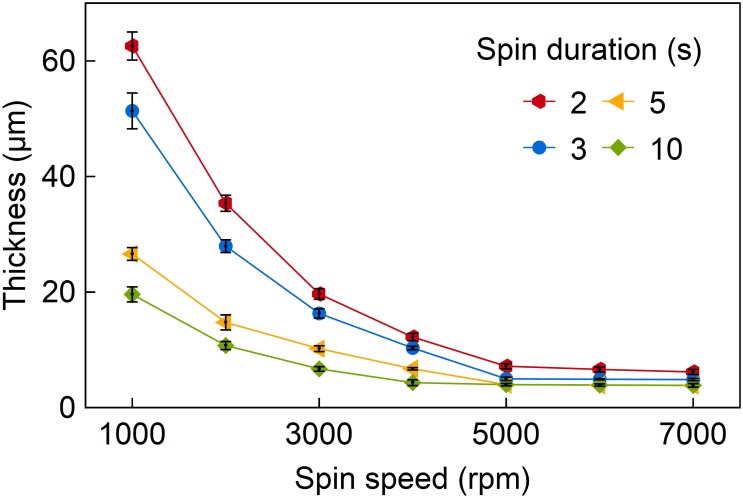
Spin-coating curve of epoxy resin E301 with the achieved layer thickness as a function of spin speed for different spin durations. A minimum layer thickness of 3.8 μm is achieved.

**Figure 5** shows the achieved epoxy resin layer thicknesses as a function of spin speed for different spin durations. For all samples the curing procedure mentioned earlier was followed. Wafers with spin durations longer than 10 s showed a highly sticky surface. They were rejected because the stickiness indicates a partial separation of the two E301 components inhibiting the complete curing of the material. In case of the test samples coated with spin durations of 2, 3, 5, and 10 s at different spin speeds epoxy resin films with a non-sticky surface and thicknesses between 3.8 and 62.5 μm are achieved. Layers thinner than 3.8 μm could not be obtained, even for spin speeds above 5,000 rpm and independent on the spin duration.

#### Cross-Linking

**FIGURE 6:**
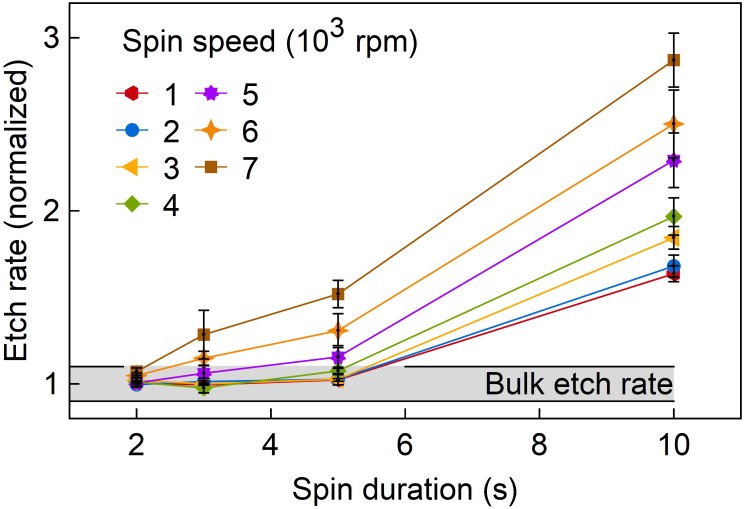
Normalized etch rate in oxygen plasma of differently spin-coated E301 thin films as a function of spin duration and spin speed. The black horizontal line indicates the minimum and maximum measured etch rates of bulk epoxy material.

**Figure 6** shows the epoxy layer etch rate in oxygen plasma normalized to the bulk etch rate of 520 ± 20 nm/min as a function of the spin duration for various spin speeds. A clear increase of the etch rate with longer spin durations as well as for higher spin speeds is found. Obviously, the highest etch rates are achieved for a spin duration of 10 s independent on spin speed. This indicates the lowest cross-linking rate as already observed with the sticky epoxy layer surfaces for spin durations longer than 10 s.

In addition, wafers coated with long spin durations and high spin speeds showed larger deviations of the etch rate across the wafer. The highest etch rates were observed towards the wafer edge. The observed correlation between spin-coating parameters and etch rates indicates that centrifugal forces separate the epoxy resin components from each other which leads to an incomplete cross-linking of the E301 layers. This separation occurs as component A has a density of ρ_A_ = 1.15 g/cm^3^ while the density of component B is ρ_B_ = 0.87 g/cm^3^ (Epoxy Technology). In conclusion, monitoring the etch rate of epoxy resin thin films represents a practicable way to compare layer properties with a bulk E301 reference sample. It allows to judge whether there is a significant separation of epoxy components reducing the cross-linking ability of the mixture.

#### Metallization

**FIGURE 7:**
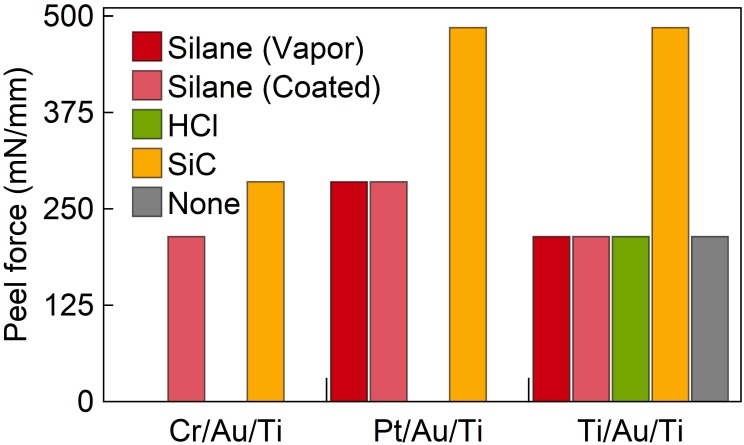
Adhesion strength obtained by peel-off tests of various metallization layer stacks deposited on epoxy and combined with different adhesion promoting layers.

This study tested three different metal layer stacks, i.e., Cr/Au/Ti, Pt/Au/Ti, and Ti/Au/Ti, and combined them with four adhesion promoting processes, namely surface silanization (vapor phase and spin-coating), epoxy resin exposure to HF, and deposition of a SiC layer. The results are summarized in **Figure 7**. The given peeling force correlates with the peeling strength of the applied adhesive tapes, i.e., Tape 1 to Tape 5.

Obviously, SiC provides the best layer adhesion independent on which metal stack is used. Following a study by Ordonez et al. (2012b), this result was expected for Pt but is now established for Cr and Ti as well. The silanization approach was best in case of the Pt/Au/Ti stack, but still lower in adhesion strength than the films on SiC. Surface modification of the functional epoxide group by exposure to HF shows no increase in adhesion for any of the tested metal stacks.

Due to the fact that Ti is easier to structure by wet and dry etching than Pt, and that it is also used as the upper metal layer, the decision was to take Ti in the form of the Ti/Au/Ti sandwich in order to ensure the adhesion on E301.

#### Sacrificial Aluminum Release

**FIGURE 8:**
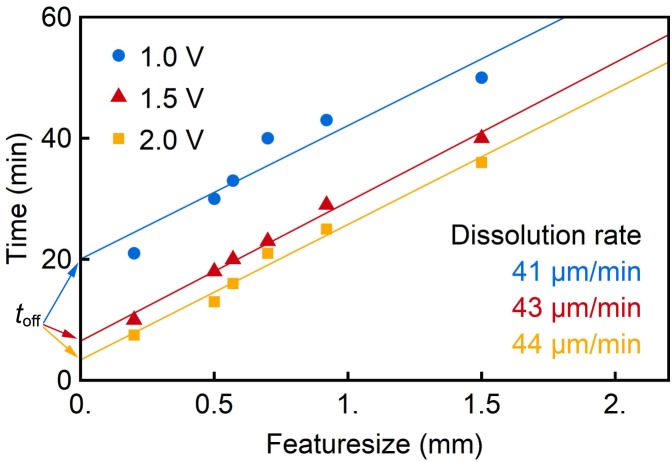
Underetch time as a function feature size for different potentials applied for the anodic dissolution of the sacrificial Al layer.

Investigating the sacrificial Al removal showed that the absolute dissolution time for different structure sizes linearly depends on the feature size to be released, as demonstrated in **Figure 8**. The linearity indicates that the process is not diffusion limited as would be expected in a purely chemical etching process where reaction products must be removed by diffusion. The dissolution rate investigated depends only on the current flow and therefore on the applied voltage. The temporal offset *t*_off_, shown in **Figure 8**, originates from Al in the 40-μm-wide etch trenches between individual probes. It needs to be dissolved before the dissolution of the Al underneath the oCI structures starts. As soon as these trenches are opened, the probe underetching proceeds until the samples are released.

**FIGURE 9:**
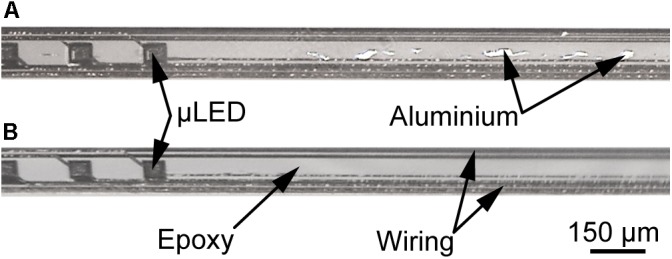
Rear of released epoxy-only oCI probe: **(A)** directly after removal of sacrificial Al layer leaving residual Al traces on the epoxy, and **(B)** after cleaning the epoxy surface for 10 min in 1 M KOH.

After successful release, some Al residues were found on the rear of the probes, as shown in **Figure 9A**. It is assumed that the dissolution process of Al depends on the release rate along grain boundaries. This can lead to Al islands losing their electrical contact to the underlying WTi layer. This stops their further dissolution. To remove these Al residuals, probes are etched in 1 M potassium hydroxide (KOH) for 10 min, which results in a clean probe **(Figure 9B)**.

### oCI Characterization

**FIGURE 10:**
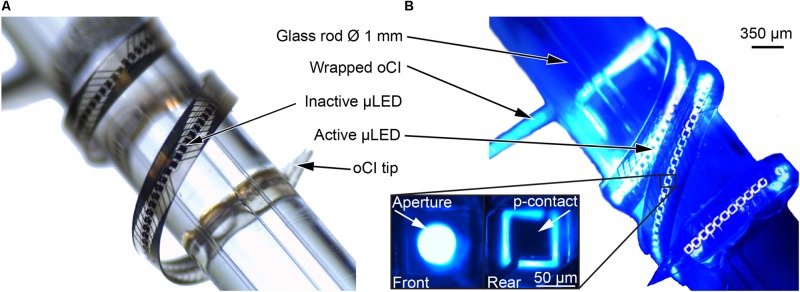
oCI sample with 144 μLEDs wrapped around a glass rod (diameter 1 mm) to demonstrate the probe capability to penetrate the curved cochlea: Micrographs taken **(A)** in daylight and **(B)** with all μLEDs operated with 1 mA, demonstrating the μLED integrity after the probe has been bent to a radius of 500 μm. Inset illustrates the μLED front and rear side with circular aperture and square p-contact, respectively.

Based on the process parameters identified in this study, oCI probes with 144 μLEDs were realized as hybrid oCIs based on a PI substrate and as epoxy-resin-only probes using three layers of E301. The example of an epoxy-resin-only probe is shown in **Figure 10**. All 144 μLEDs can be addressed and operated as indicated on the right-hand side of **Figure 10**. To demonstrate the ability of the oCI to wind into a cochlea, it was successfully wrapped around a glass rod with a diameter of 1 mm, resulting in a bending radius of 500 μm.

#### Thermomechanical Behavior

**FIGURE 11:**
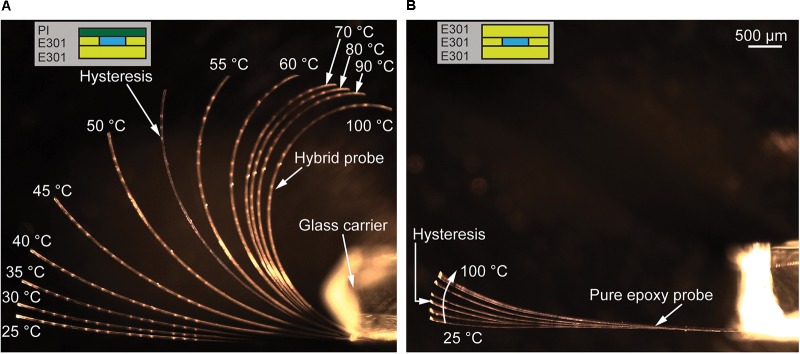
Thermomechanical behavior of an oCI probe during a heating/cooling cycle between RT and 100°C. **(A)** Hybrid oCI probe with a maximum bending of more than 180° and a hysteresis of approximately 90° when returning to RT; **(B)** pure epoxy oCI probe with minimal bending and a hysteresis below 10°. The insets in **(A)** and **(B)** illustrate probe cross-sections with μLED indicating the layer orientation relative to the glass carrier.

The overarching aim of the technical developments described here is the optimization of the thermomechanical behavior of oCI probes. This behavior is determined by the different CTEs of the applied materials, namely PI and the epoxy resin E301 (Goßler et al., 2014), causing thermomechanical stress in the oCI and probe bending. We therefore compare a hybrid oCI probe with an epoxy-only sample by varying the temperature between RT and 100°C. **Figure 11** shows probe side views of both probe variants taken at several temperatures when increasing the temperature from RT and cooling back from 100°C to RT. The remaining probe bending indicates a mechanical hysteresis in the thermomechanical probe response. As shown in **Figure 11A**, the hybrid oCI bends toward the PI side by more than 180° resulting in a bending radius of 1.65 mm. Already at 80°C, the probe tip points in the opposite direction compared to the original orientation at RT. In contrast, the pure epoxy oCI just bends slightly within the same temperature interval. This bending is due to a small variation in the thickness of the first and third polymer layers of the oCI, caused by applying the same spin-coating parameters for both layers but having different surfaces. After cooling both oCIs down to RT, a hysteresis is observed, which is likely caused by the glass transition temperature of E301 around 55°C. In conclusion, irreversible bending is induced into the oCI. In the case of the hybrid oCI, this hysteresis deflects the tip by about 90°. This is a significant challenge when trying to implant the probe into the cochlea: in fact a probe curling into the opposite direction would be required for the μLED to directly face the SGNs. The increased CTE of E301 above its glass transition temperature is reflected in the increased bending between 40 and 60°C, as shown in **Figure 11A**. The pure epoxy oCI shows only a small hysteresis. It is deflected by about 10° from its original position, which is still suitable for probe implantation into the cochlea.

#### Thermal Characterization

**FIGURE 12:**
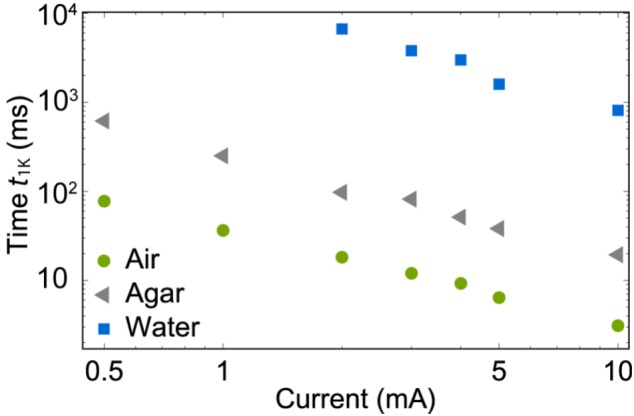
Time *t*_1K_ needed to increase the probe surface temperature by 1 K as a function of the applied μLED DC current with the oCI probe floating in air (circles) or being immersed in agarose gel (triangles) or water (squares).

**Figure 12** shows the time *t*_1K_ needed to increase the probe temperature by 1 K as a function of the μLED DC current varied between 0.5 and 10 mA and the material surrounding the probe. As expected, the surface temperature is strongly dependent on the immersion material, i.e., the fastest temperature increase is observed when the probe is floating in air. In this case, the 1 K-limit is already reached within *t*_1K_ = 3 ms at a maximum μLED current of 10 mA. In contrast, due to the high thermal conductivity of water of ca. 0.62 Wm^-1^ K^-1^ heat dissipation is highly increased resulting in an increased rise time *t*_1K_ of 800 ms. In contrast, a value of *t*_1K_ = 11 ms is obtained for the agarose gel immersed probe applying a DC current of 10 mA. This faster probe heating is presumably caused by a reduced convection in agarose gel compared to water.

#### Optical Characterization

**FIGURE 13:**
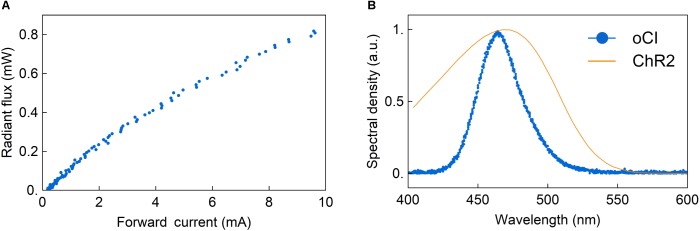
**(A)** Optical power vs. drive current of a μLED of an epoxy-only oCI. The measurement is performed at 10 kHz and a duty cycle of 10%; an emittance of 407 mW/mm^2^ is achieved at 10 mA for an μLED aperture with a diameter of 50 μm. **(B)** Representative normalized spectral density vs. wavelength of μLEDs on epoxy-only oCI probes with a peak at 462 nm (circles) compared to the optical sensitivity of ChR2 (solid line) (Nyns et al., 2017).

The optical characterization of the integrated μLED using the integrating sphere revealed a radiant flux of a single μLED of 0.82 mW at a forward current of 10 mA, as shown in **Figure 13A**. This corresponds to an emittance of 407 mW/mm^2^, referred to an emitting surface with a diameter of 50 μm. A slightly non-linear response is obtained, indicating a drop of the μLED efficiency caused by thermal and non-thermal effects. The thermal response is increased due to the fact that the oCI probe was operated in air and was thus thermally well isolated. As demonstrated by Schwaerzle et al. (2016) and replicated in our thermal experiments, operating an LED-based probe in contact with agarose gel taking the role of a heat sink, the temperature increase can be minimized, thus optimizing the LED efficiency. As indicated in **Figure 13B**, the peak wavelength is at 462 nm, which is suitable for the stimulation of ChR2.

## Conclusion

This work developed and validated a new fabrication process of optical CIs based on a single biocompatible and optically highly transparent epoxy resin used as substrate and passivation materials. We substituted the PI substrate initially used by Goßler et al. (2014) by the epoxy resin E301. This development was motivated by the fact that the existing hybrid oCI probe variant is hampered by a pronounced residual and thermomechanical probe bending due to the CTE difference of the implemented materials.

The process development included a dedicated spin-coating procedure of the solvent-free epoxy resin E301 to guarantee layers of homogeneous thickness. Optimizing two spin-coating parameters, namely spin speed and spin duration, we reliably achieved layer thicknesses between 3.8 and 62.5 μm. In order to prevent the uncured films from collapsing into E301 islands an appropriate adhesion promoter has been introduced in combination with a fast curing procedure applied immediately after spin-coating. Epoxy layers with a nearly perfect surface and homogenous thicknesses with a maximum variation of 7% across 4-inch wafers were achieved.

Since the cross-linking of the epoxy layers strongly depends on the spin-coating parameters, the process window was analyzed using a plasma-based dry etching process of the epoxy resin layers. It was introduced in this study to replace differential scanning calorimetry which cannot be applied in the case of thin-film materials spin-coated to carrier wafers. Depending on the spin speed and spin duration, we observed etch rates increased by a factor of up to 3 compared to optimally cured E301 bulk samples. Fully cured epoxy layers with etch rates similar to bulk material were achieved for a variety of spin speed and spin duration combinations enabling a wider range of layer thicknesses. As an example, the thinnest epoxy layer with a thickness of 5 μm that could be cured completely was achieved at 5,000 rpm and with a spin duration of 3 s. On the other hand, long and fast spin-coating resulted in a separation of the two components of E301. This resulted in an incomplete curing, as indicated by the increased etch rate in the oxygen plasma.

In order to guarantee the probe integrity, we optimized the layer adhesion between the μLED metallization and the underlying polymer substrate. Various metal layer compositions and adhesion promoters were tested. It was found that SiC in combination with Pt or Ti layers leads to the best results, with a peeling force of at least 485 mN/mm as extracted using a peeling test. Titanium was finally chosen for the oCI metallization as it can be structured by wet and dry etching and as it has already been established as the upper layer of the metallization stack.

The probe release of the epoxy-only probes required in addition the introduction of a structured sacrificial Al layer. It is deposited on a permanent WTi layer which provides the electrical contact during the anodic metal dissolution. The dissolution time was found to be linearly dependent on the distance to be undercut on the oCI wafer. The absolute time to lift an oCI using this technique was determined to be 23 min. In contrast to the peel-off approach used for PI layers on SiO*_x_*, the method has the clear advantage of allowing a stress-free probe release. From the process compatibility point of view, the peel-off should also be considered to be used for PI-based ribbon cables used for Si-based neural implants (Kisban et al., 2009), electrocorticography (ECoG) electrode arrays (Rubehn et al., 2009) or cuff-electrodes (Schuettler et al., 2000).

The study presented here compared two oCI variants, i.e., a conventional hybrid probe based on PI and epoxy resin as well as an epoxy-based device composed of a three-layer epoxy stack. We demonstrated that the epoxy-only variant is definitely superior as far as its thermomechanical response is concerned. The epoxy-only probe shows minimal bending and a strongly reduced thermomechanical hysteresis. This is a clear advantage for probe implantation into the cochlea.

The thermal probe characterization revealed that the temperature increase can be safely limited to 1 K depending on the μLED DC current, the stimulation duration, and the medium surrounding the μLED probe. Using agarose gel as a tissue phantom, the 1 K-limit is reached within ca. 11 ms applying a DC current of 10 mA. In contrast, a stimulation duration of 800 ms is needed to reach this temperature increase when the probe is immersed into water. It has to be kept in mind that it is yet unclear whether the medium inside the cochlea can be thermally modeled by agarose gel or liquid water as done in our tests. In comparison to pulse lengths below 10 ms, as typically applied *in vivo* (Hernandez et al., 2014), the temperature increase of our μLEDs can in any case be safely limited to 1 K.

The realized oCI probe with 144 individually controllable μLEDs offers radiant flux and emittance values of 0.82 mW and 407 mW/mm^2^ per μLED at DC currents of 10 mA, respectively, well exceeding the optical threshold of 1–4 mW/mm^2^ (Boyden et al., 2005; Deisseroth et al., 2006). With a peak wavelength of 462 nm and the given emittance, the optical probe reported here perfectly matches the requirements of optogenetic neuronal experiments based on ChR2.

The technology presented in this study can easily be transferred to other fields of optogenetic applications where probe flexibility and highly resolved μLED patterns are needed. As an example, it is conceivable to temporarily stiffen the linear arrays presented here using a biodegradable material (Boyden et al., 2005; Gilgunn et al., 2012) or an inserter tool (Barz et al., 2015; Felix et al., 2013), enabling a probe implantation into cortical tissue. Once implanted, the highly flexible probe will be beneficial in view of a long-term applications at minimal tissue reaction. Similarly, flexible two-dimensional (2D) μLED arrays realized using our technological approach will provide a high lateral resolution to stimulate the brain tissue while the probe itself adapts to the corrugated brain surface at minimal bending forces. A combination of 2D μLED arrays with state-of-the-art ECoG electrodes arrays (Kozai and Kipke, 2009) is also foreseeable providing simultaneously highly resolved optogenetic stimulation and electrophysiological recordings (Stieglitz et al., 2009). Aside from basic neuroscientific research, we are convinced that the technology is applicable as well in the field of cardiology, i.e., as optogenetic cardiac pacemaker or defibrillator (Kwon et al., 2013b; Bruegmann et al., 2016; Crocini et al., 2016; Diaz-Maue et al., 2018).

## Author Contributions

EK processed the oCIs, developed the epoxy thin-film process, performed the experiments, analyzed the measurement data, prepared the figures, and contributed the manuscript. EK and CG developed the oCIs and realized the photolithography masks. OP and PR discussed the technical concepts and experimental results, and finalized the manuscript. All authors reviewed the manuscript.

## Conflict of Interest Statement

The authors declare that the research was conducted in the absence of any commercial or financial relationships that could be construed as a potential conflict of interest.

## Footnotes

1 http://www.epotek.com/site/administrator/components/com_products/assets/files/Style_Uploads/301.pdf
